# Diagnostic accuracy of serum calprotectin measured by CLIA and EIA in juvenile idiopathic arthritis: a proof-of-concept study

**DOI:** 10.3389/fped.2024.1422916

**Published:** 2024-06-19

**Authors:** Helena Codes-Méndez, Berta Magallares-López, Hye-Sang Park, Anaís Mariscal, Cándido Juárez, Susana Boronat, Laura Martínez-Martínez, Hector Corominas

**Affiliations:** ^1^ Rheumatology Department, Hospital de la Santa Creu I Sant Pau, Barcelona, Spain; ^2^ Department of Medicine, Institut de Recerca Sant Pau (IR SANT PAU), Barcelona, Spain; ^3^ Universitat Autònoma de Barcelona, Bellaterra, Spain; ^4^ Immunology Department, Hospital de la Santa Creu I Sant Pau, Barcelona, Spain; ^5^ Pediatric Department, Hospital de de la Santa Creu I Sant Pau, Barcelona, Spain

**Keywords:** juvenile idiopathic arthritis, serum calprotectin, biomarkers, s100 protein, s100A8/S100A9, calcium-binding myeloid protein p8/14

## Abstract

**Objective:**

C-reactive protein (CRP) and erythrocyte sedimentation rate (ESR) are used to assess disease activity in juvenile idiopathic arthritis (JIA). However, because these biomarkers do not always differentiate between active and inactive disease, there is a need for alternative markers such as serum calprotectin (sCal). The main aim of this proof-of-concept study was to assess the diagnostic accuracy of sCal in patients with JIA. Secondary aims were to identify the optimal sCal cut-off levels to define active disease and evaluate the association between these biomarkers and disease activity status.

**Methods:**

Serum samples were obtained from 25 pediatric patients with JIA. Serum calprotectin levels were determined by two different assays, the QUANTA FLASH chemiluminescence immunoassay (CLIA) from Inova Diagnostics and the solid-phase enzyme immunoassay (EIA) from Bühlmann Laboratories. Diagnostic accuracy was assessed for sCal CLIA, sCal EIA, CRP, and ESR. The results obtained by the CLIA and EIA methodologies were compared. We also evaluated the association between the individual each biomarkers (sCal CLIA, sCal EIA, CRP, and ESR) and disease activity (according to JADAS-27 criteria and the ACR criteria modified by Anink and colleagues).

**Results:**

For both sCal assays (CLIA and EIA), the optimal cut-off level (ROC analysis) was the same (2.3 µg/ml). Serum calprotectin levels measured by CLIA and EIA were strongly correlated with each other (Kendall's tau-b, 0.71; *p* < 0.001). Compared to ESR and CRP, sCal CLIA and EIA were both more accurate (i.e., greater sensitivity) in identifying patients with active disease. By contrast, ESR and CRP were more effective in identifying patients in remission (i.e., better specificity).

**Conclusion:**

This proof-of-concept study shows that determination of serum calprotectin levels with CLIA or EIA can accurately identify the presence of active disease in patients with JIA.

## 1 Introduction

Juvenile idiopathic arthritis (JIA) is a group of diseases characterized by arthritis of unknown etiology persisting for at least 6 weeks, with onset occurring before age 16 (1). It is the most common childhood inflammatory rheumatic condition, with an estimated annual incidence of 7.8/100.000 (1, 2). According to the International League of Associations for Rheumatology (ILAR), there are seven differing subtypes of JIA: (1) oligoarthritis; (2) rheumatoid factor (RF)-positive polyarthritis; (3) RF-negative polyarthritis; (4) enthesitis-related arthritis (ERA); (5) psoriatic arthritis (PsA); (6) systemic arthritis; and (7) undifferentiated arthritis (3). Although the ILAR criteria are still considered the standard, it is worth noting that several groups have sought to revise these criteria in recent years (4, 5).

Due to the highly heterogenous nature of JIA—as evidenced by the seven different subtypes and by the controversy surrounding the classification system—it has been challenging to identify simple measures that can be used to monitor the course of the disease, establish prognosis, and predict the likelihood of disease progression into adulthood (6). The management of JIA depends on a range of different factors, including the number of affected joints, the presence of uveitis, sacroiliitis or enthesitis, and laboratory biomarkers, including positive antinuclear antibodies, positive rheumatoid factor, and anti-citrullinated protein antibodies (7–9). Conventional acute phase reactants such as C-reactive protein (CRP) and erythrocyte sedimentation rate (ESR) are widely used to assess inflammatory status in JIA, and both of these measures are key components of composite disease activity indices, including the Juvenile Arthritis Disease Activity Score (JADAS-27) (10, 11). However, these biomarkers—or indices based on them—may not be accurately differentiate between patients with active and inactive disease, especially in patients with oligoarthritis (4, 6). In fact, patients commonly present normal ESR and/or CRP values despite clinical signs of active disease. Despite these limitations, both of these measures are good predictors of disease progression (6, 9, 12).

Given the limitations of ESR and CRP in JIA, numerous authors have proposed serum calprotectin (sCal) as an alternative biomarker of inflammatory activity (6, 12–18). Serum calprotectin levels have been shown to correlate well with disease activity—especially in systemic JIA—and the available data suggests that sCal is a more specific biomarker of disease activity than either ESR or CRP (19–22). Several different commercial tests are available to determine sCal levels, including chemiluminescence immunoassay (CLIA), solid-phase enzyme immunoassay (EIA), and enzyme-linked immunosorbent assay (ELISA). However, the diagnostic accuracy of these tests—that is, their capacity to distinguish between active and inactive disease in JIA—has not been well-established. Similarly, the optimal cut-off point (sCal level) to determine the presence of disease activity in patients with JIA has not been established. In addition, to our knowledge, these different methods of determining sCal levels in JIA have not been compared to date.

In this context, the present proof-of-concept study had two main aims: 1) to assess the diagnostic accuracy of serum calprotectin as a biomarker of disease activity status in patients with JIA, and 2) to determine the optimal sCal cut-off values, as measured by CLIA and EIA, to differentiate between active and inactive disease. In addition, we also sought to determine the association between these biomarkers (sCal CLIA, sCal EIA, CRP, and ESR) and disease activity.

## 2 Materials and methods

### 2.1 Study population

This was a cross-sectional study carried out at a pediatric rheumatology outpatient clinic at a tertiary referral hospital in Barcelona, Spain.

All patients who met ILAR criteria for JIA (3) were consecutively included from June 2020 through June 2021. The patients (*n* = 25) were classified into four clinically-homogeneous groups based on the 2019 American College of Rheumatology/Arthritis Foundation guidelines for the treatment of JIA (8) and on the 2019 Printo criteria (4). The four groups were as follows: (1) systemic JIA (same criteria as ILAR systemic JIA); (2) oligoarticular JIA (non-systemic arthritis with <5 joints ever involved); (3) polyarticular JIA (non-systemic arthritis with ≥5 joints ever involved); and (4) enthesitis/spondylitis-related JIA (sacroiliitis or enthesitis-related arthritis). Serum samples (*n* = 25) were collected from the 25 patients as part of routine clinical practice. Patients with other concomitant diseases such as cancer or chronic infectious diseases that might interfere with biomarker interpretation were excluded.

The study was approved by the institutional ethics committee of Hospital de la Santa Creu I Sant Pau (code: IIBSP-BIR-2017-07). The study was conducted in accordance with the Helsinki Declaration.

### 2.2 Data collection and study variables

Patient data were obtained from the hospital's electronic healthcare records. Demographic data (sex and date of birth) and disease-related variables, including JIA subtype classification, were also collected. The following clinical variables were also recorded: number of joints with active disease based on physical examination (active joint count), JADAS-27 score, laboratory parameters, and prescribed treatment.

Serum calprotectin levels were measured by both CLIA and EIA. CRP and ESR were measured as part of routine clinical practice and used as a benchmark for comparison with the sCal values.

Disease activity was assessed by two different sets of criteria, those described by Anink et al. (18) and JADAS-27 (10). First, we used the modified interpretation of the American College of Rheumatology (ACR) pediatric response criteria term “inactive disease” as defined by Anink et al. (18), referred to in the manuscript as “ACR-modified” criteria.

Those authors defined inactive disease as follows: no active arthritis; absence of systemic features and uveitis; normal ESR (≤ 20 mm/h), and physician's global assessment (PGA) indicating absence of disease activity (score ≤10 on a 0–100 mm scale). We chose to use the ACR-modified definition of disease inactivity because it is consistent with the findings of recent studies that have investigated sCal levels in patients with JIA (23). Furthermore, this definition is more comprehensive than the JADAS-27 scale, as it covers a broader spectrum of disease by including eye involvement (uveitis), and it also accommodates a more pragmatic PGA (higher cut-off), which makes it more feasible in the context of routine clinical practice. Second, we applied the inactive disease criteria from JADAS-27 (10), which is the standard approach for assessing disease activity in patients with JIA. However, JADAS-27 has an important limitation: disease activity is mainly based on the presence of clinical arthritis. The JADAS-27 score was categorized as an ordinal variable in order to group patients into four different levels of disease activity, as follows: inactive, low, moderate, and high. The specific cut-off levels varied according to the JIA subtype (24).

We evaluated the diagnostic accuracy—defined as the test's ability to differentiate between inactive and active disease—of sCal measured by CLIA and EIA. Disease activity was assessed according to the ACR-modified criteria and JADAS-27.

### 2.3 Determination of serum biomarkers

Blood samples were collected from one to seven days before the clinical evaluation. Serum calprotectin was measured by the QUANTA Flash® CLIA assay (Inova Diagnostics) and by solid-phase EIA (Bühlmann Laboratories AG). Given that serum sCal concentrations may increase significantly if centrifugation is performed ≥6 h after extraction (21) (neutrophils in the serum continue to release sCal even after sample collection), strict preanalytical test conditions were established and all serum samples were centrifuged and stored at −80°C within 2 h of extraction.

For the evaluation of ESR and CRP, the upper limit for normal levels was set at 20 mm/h for ESR and 5 mg/L for CRP.

### 2.4 Statistical analysis

Descriptive statistics are presented as absolute frequencies with medians and interquartile range (IQR) or as means with standard deviation (SD), as appropriate. The Shapiro-Wilk test and box plots were used to check the distribution of the variables.

The cut-off levels for sCal (CLIA and EIA), CRP, and ESR were evaluated according to both JADAS-27 and ACR-modified criteria using receiver operating characteristic (ROC) curves. Optimal cut-off values were based on the area under the curve (AUC), sensitivity, specificity, and the likelihood ratio.

The association between each biomarker (sCal CLIA, sCal EIA, ESR, CRP) and disease activity (assessed by JADAS-27 and ACR-modified criteria) was evaluated. Odds ratios (OR) for these associations were determined for each biomarker through logistic regression analysis. Finally, a multinomial logistic regression model was used to evaluate the relationship between each sCal determination technique (CLIA and EIA) and disease activity status based on JADAS-27 score. Two-sided 95% confidence intervals (CI) were calculated. Statistical significance was set at *p* < 0.05. Stata 16.0 (Statacorp, College Station, TX, USA) was used to perform all statistical analyses.

## 3 Results

### 3.1 Baseline clinical characteristics and biomarker distribution according to disease activity status

A total of 25 patients diagnosed with JIA (ILAR criteria) (3) were included in the study. The clinical and serological characteristics of these patients are shown in Table 1.

**Table 1:** Patient characteristics according to diagnostic criteria (ACR-modified vs. JADAS-27).

Disease activity score	Total (*n* = 25)	ACR-modified criteria		JADAS-27	
Variable		Active disease (*n* = 12)	Inactive disease (*n* = 13)	Active disease (*n* = 11)	Inactive disease (*n* = 14)
Female, *n* (%)	12 (48)	6 (50)	6 (46.2)	5 (45.4)	7 (50)
Mean age, years (SD)	11.42 (4.6)	11.84 (4.96)	11.03 (2.95)	12.9 (5.6)	10.3 (3.4)
Mean disease duration, years (SD)	2.21 (2.7)	1.42 (2.9)	2.95 (2.4)	1.7 (2.99)	2.6 (2.5)
JIA subtype, *n* (%)					
Oligoarticular	11 (44)	6 (50)	5 (38.5)	5 (45.4)	6 (42.9)
Polyarticular	5 (20)	4 (33.3)	1 (7.7)	4 (36.4)	1 (7.1)
Enthesitis/Spondylarthritis	7 (28)	2 (16.7)	5 (38.5)	2 (18.2)	5 (35.7)
Systemic	2 (8)	0 (0)	2 (15.4)	0 (0)	2 (14.3)
sCal EIA, µg/ml					
p50 (IQR)	3.1 (0.8–8.1)	3.17 (1.3–8.1)	2.91 (0.8–5.3)	3.26 (1.9–8.1)	2.7 (0.8–5.3)
Mean (SD)	3.4 (1.8)	3.97 (2.1)	2.79 (1.3)	4.2 (2.1)	2.7 (1.3)
sCal CLIA, µg/ml					
p50 (IQR)	2.3 (0.1–6.6)	2.6 (0.1–6.6)	1.9 (1.1–5.2)	2.8 (0.1–6.6)	2.1 (1.1–5.2)
mean (SD)	2.9 (1.7)	3.2 (1.97)	2.6 (1.5)	3.3 (2.0)	2.6 (1.5)
CRP, mg/L					
p50 (IQR)	1.6 (0.5–17.5)	10.8 (0.5–4.7)	10.8 (0.5–4.7)	4.8 (0.5–17.5)	0.75 (0.5–4.7)
Mean (SD)	3.1 (3.9)	5.00 (4.97)	1.28 (1.2)	5.4 (5)	1.22 (1.1)
ESR, mm/h					
p50 (IQR)	9 (1–64)	15 (2–64)	8 (1–19)	18 (2–64)	8 (1–19)
Mean (SD)	13.6 (14.1)	19.41 (18.1)	8.2 (5.6)	20.5 (18.6)	8.1 (5.4)
Active joint count, p50 (IQR)	0 (0–17)	1.5 (0–17)	0 (0–0)	2 (0–17)	0 (0–1)
Active treatment, *n* (%)					
Prednisone	5 (20)	4 (33.3)	1 (7.7)	2 (18.2)	3 (21.4)
cDMARD	13 (52)	9 (75)	4 (30.8)	7 (63.6)	6 (42.9)
bDMARD	6 (24)	1 (8.3)	5 (38.5)	2 (18.2)	4 (28.6)

SD, standard deviation; sCal, serum calprotectin; IQR, interquartile range; CRP, C-reactive protein; ESR, erythrocyte sedimentation rate; cDMARD, conventional disease-modifying antirheumatic drugs; bDMARD, biological disease-modifying antirheumatic drugs.

According to JADAS-27 criteria, 11 patients (44%) had active disease, distributed by activity level as follows: low (*n* = 6; 56%), moderate (*n* = 1, 9.1%), and high (*n* = 4; 36%). The remaining 14 patients were considered to have inactive disease.

Two patients developed uveitis after diagnosis. At the time of data collection, both of these patients were considered to have active disease according to ACR-modified criteria, but only one was active based on the JADAS-27 criteria.

#### 3.1.1 Treatment

Six patients received prednisone (mean daily dose, 4.5 mg). Thirteen patients were treated with conventional disease-modifying antirheumatic drugs (cDMARDs). Of these, 8 (61.5%) received methotrexate, 4 (30.7%) received leflunomide, and 1 (7.7%) received sulfasalazine (Salazopyrine). Six patients received biological DMARDs (bDMARDs), as follows: anakinra and tocilizumab (*n* = 2 patients with systemic JIA); adalimumab (*n* = 2, enthesitis-related arthritis JIA); and etanercept and abatacept (*n* = 2, polyarticular JIA).

**Figure 1:**
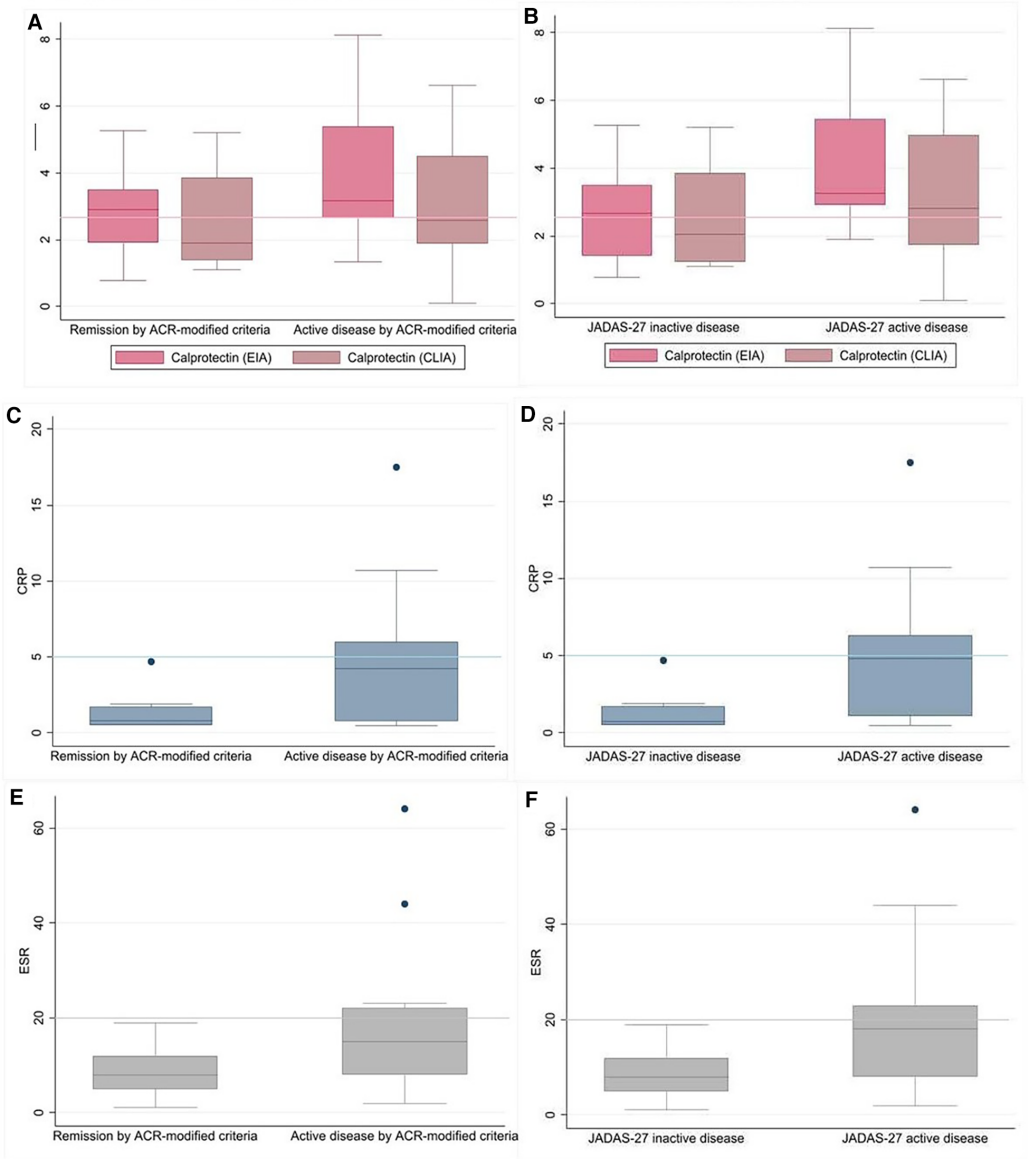
Distribution of biomarkers according to disease activity subgroup. Serum calprotectin (sCal) levels measured by QUANTA flash ® CLIA and Bühlmann® EIA and depending on disease activity state as assessed by ACR-modified criteria (**A**) of JADAS-27 (**B**), with the cut-off value of 2.3μg/ml represented in a line. Serum concentrations of CRP in inactive and active disease by ACR-modified criteria (**C**) and JADAS-27 (**D**), with a cut-off level of 5 mg/L. Serum ESR levels by disease activity subgroups by ACR-modified criteria (**E**) and JADAS-27 (**F**), with a cut-off value of 20 mm/h.

The distribution of serum biomarker concentrations based on disease activity is shown in Figure 1. As that figure shows, the thresholds for these biomarkers were within the IQR for the subgroup with active disease. In the patients with active disease (ACR-modified and JADAS-27), sCal levels measured by EIA surpassed the 2.3 µg/ml cut-off in >75% of cases (Figures 1A,B); by contrast, sCal CLIA, CRP, and ESR levels only exceeded their respective cut-off values in <50% of cases with active disease (Figures 1C–F). Finally, CRP and ESR had a greater capacity than sCal (regardless of the specific technique, CLIA or EIA) to identify patients with inactive disease.

A contingency table showing the true-positive, true-negative, false positive, and false-negative counts for inactive disease status (according to the proposed cut-off values) is shown in the supplementary material (Supplementary Table S1).

### 3.2 Diagnostic accuracy of sCal levels measured by CLIA and EIA

We evaluated the diagnostic accuracy of sCal CLIA, sCal EIA, CRP, and ESR, with the latter two measures used as the reference standard.

**Figure 2:**
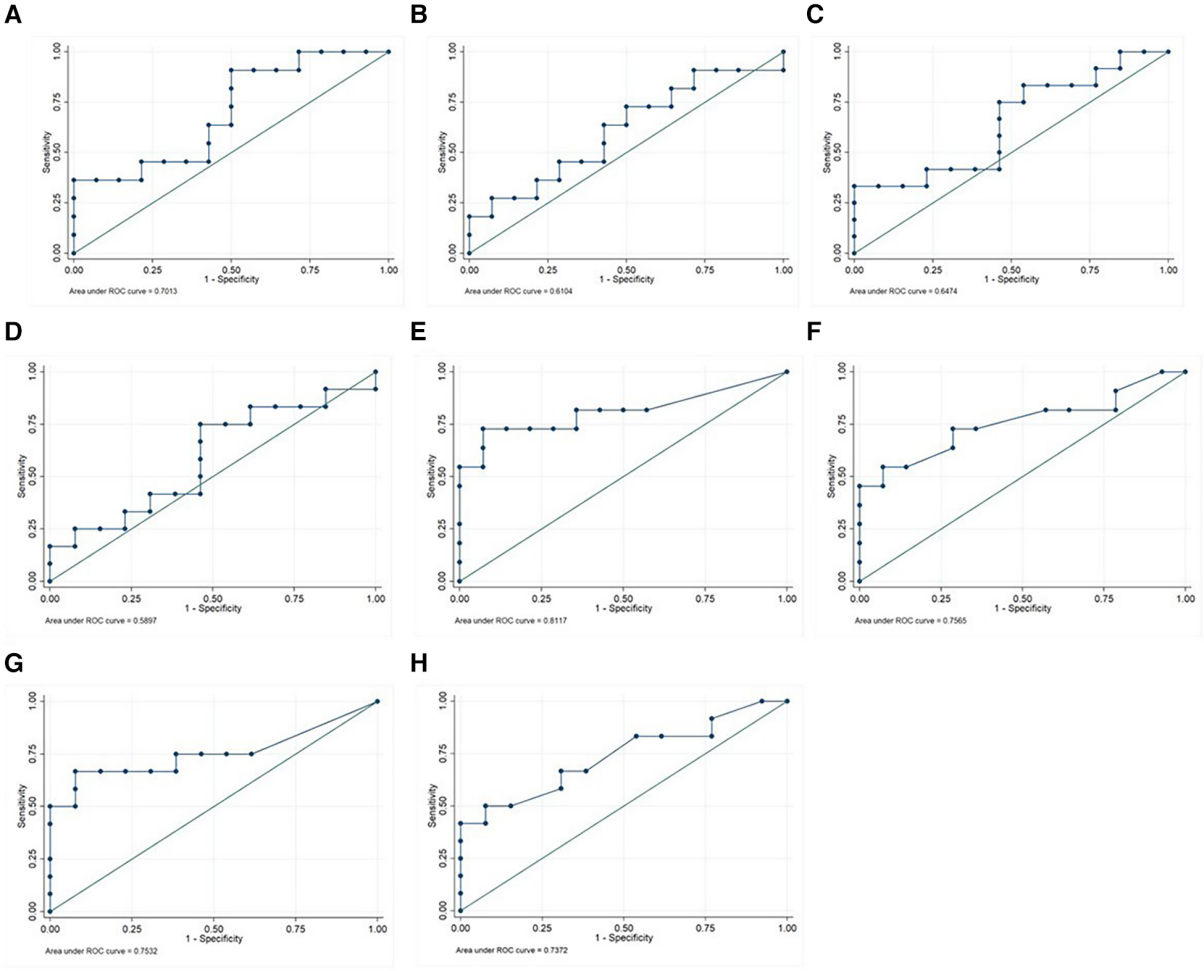
ROC curve analysis of sCal CLIA, sCal EIA, CRP, and ESR for the differentiation between active and inactive disease according to ACR-modified criteria and JADAS-27. ROC curves of sCal EIA (**A**), sCal CLIA (**B**), CRP (**E**) and ESR (**G**) for disease activity by JADAS; and ROC curves of sCal EIA (**C**), sCal CLIA (**D**), CRP (**F**) and ESR (**H**) for disease activity by ACR-modified criteria are shown. ROC: receiver operating characteristic; sCal: serum calprotectin. CRP: C-reactive protein. ESR: erythrocyte sedimentation rate.

Tables 2, 3 show the proposed cut-off values with the diagnostic performance (maximum efficiency and ROC curves) to predict disease activity according to the ACR-modified and JADAS-27 criteria, respectively. Optimal cut-off levels for each biomarker were identified using ROC curves (Figure 2), and cut-off levels for maximum efficiency were also determined.

**Table 2:** Performance of the four biomarkers based on ACR-modified criteria.

Variable	sCal EIA, µg/ml	sCal CLIA, µg/ml	ESR, mm/h		CRP, mg/L
AUC (95% CI)	0.64 (0.42–0.82)	0.58 (0.38–0.78)	0.73 (0.50–0.87)		0.75 (0.54–0.90)
Cut-off for maximum efficiency	5.3	2.0	20	18	3.1
* *Sensitivity	33.3%	75%	41.7%	50%	66.7%
* *Specificity	100%	53.8%	100%	92.3%	92.3%
* *LR+	–	1.62	–	6.50	8.66
* *LR-	0.66	0.46	0.58	0.54	2.77
Cut-off based on ROC	2.9	2.0	10		3.1
* *Sensitivity	75%	75%	66.7%		66.7%
* *Specificity	53.8%	53.8%	69.2%		92.3%
* *LR+	1.62	1.62	2.16		8.66
* *LR-	0.46	0.46	0.48		0.36

AUC, area under the curve; ROC, receiver operating characteristic; LR+, positive likelihood ratio; LR-, negative likelihood ratio; sCal, serum calprotectin; CRP, C-reactive protein; ESR, erythrocyte sedimentation rate; CI, confidence interval.

**Table 3:** Performance of the four biomarkers according to JADAS-27.

Variable	sCal EIA, µg/ml	sCal CLIA, µg/ml		ESR, mm/h		CRP, mg/L
AUC (95% CI)	0.70 (0.50–0.87)	0.61 (0.38–0.78)		0.75 (0.54–0.90)		0.81 (0.59–0.93)
Cut-off for maximum efficiency	5.3	4.9	6.0	20	18	3.1
* *Sensitivity	36.4%	27.3%	18.2%	45.5%	54.5%	72.7%
* *Specificity	100%	92.9%	100%	100%	92.9%	92.9%
* *LR+	–	3.81	–	–	7.63	10.18
* *LR-	0.63	0.78	0.81	0.54	0.48	0.29
Cut-off based on ROC	2.3	2.3		10		3.1
* *Sensitivity	90.9%	63.6%		72.7%		72.7%
* *Specificity	50%	57.1%		71.4%		92.2%
* *LR+	1.81	1.48		2.54		10.18
* *LR-	0.18	0.63		0.38		0.29

AUC, area under the curve; ROC, receiver operating characteristic; LR+, positive likelihood ratio; LR-, negative likelihood ratio; sCal, serum calprotectin; CRP, C-reactive protein; ESR, erythrocyte sedimentation rate; CI, confidence interval.

Based on the suggested cut-off values for disease activity according to the ACR-modified criteria (Table 2), the optimal cut-off values were as follows: 2.0 µg/ml for sCal CLIA (positive predictive value [PPV] and negative predictive value [NPV] of 60% and 70%, respectively) and 2.9 µg/ml for sCal EIA (PPV and NPV, 60% and 70%, respectively).

The optimal threshold for sCal CLIA (2.0 µg/ml, Table 2) was the same as recommended by the manufacturer of the CLIA kit. Serum calprotectin, whether measured by EIA or CLIA, was more sensitive (75%) than CRP and ESR, although the specificity was lower. Using the 2.3 µg/ml threshold to indicate active disease (ACR-modified criteria), sCal CLIA had a sensitivity of 58.3% and specificity of 53.9% (PPV, 57.8%; NPV, 53.9%); by comparison, the sensitivity and specificity for sCal EIA was 83.3% and 46.2%, respectively (PPV, 58.8%; NPV, 75%).

Conversely, utilizing the recommended cut-off values for disease activity according to the JADAS-27 criteria, the optimal sCal cut-off value for both techniques (CLIA and EIA) was 2.3 µg/ml (Table 3). The PPV and NPV values for CLIA were 53.8% and 66.7%, respectively vs. 58.8% and 87.5% for EIA. For this cut-off value (2.3 µg/ml), the sensitivity of EIA was 90.9%, which was superior to the sensitivity obtained for CRP, ESR and sCal CLIA. For sCal CLIA, the corresponding sensitivity was 63.6%, which was lower than the sensitivity obtained for CRP, ESR, and sCal EIA. The specificity for both sCal CLIA and EIA was lower than CRP and ESR (Table 3).

For CRP and ESR, the standard cut-off values for disease activity are 5 mg/L and 20 mm/h, respectively. Using these cut-off values, both CRP and ESR had a lower sensitivity but better specificity than sCal EIA and sCal CLIA (Table 3). The AUC for CRP was 0.75 (sensitivity, 50%; specificity, 100%); for ESR, the AUC was 0.73 (sensitivity, 41.7%; specificity, 100%). The optimal cut-off values for CRP and ESR (ROC analysis) were significantly lower (3.1 mg/L and 10 mm/h, respectively) than the standard clinical thresholds. However, both CRP and ESR were less sensitive and more specific than sCal when using those lower thresholds.

### 3.3 Association between biomarkers and disease activity

#### 3.3.1 Odds ratio for inactive disease according to ACR-modified and JADAS-27 criteria

The OR was computed to assess the association between disease activity and each biomarker (Table 4). CRP showed a moderate to high association with disease activity (as measured by both ACR-modified criteria and JADAS-27); in both cases, the association was statistically significant. Serum calprotectin (EIA) showed a moderate association with disease activity (OR: 1.52 [ACR-modified] and 1.79 [JADAS-27]), but this association was not statistically significant, despite the high precision indicated by the 95% CI. By contrast, sCal CLIA showed a weaker association with disease activity (OR: 1.19 [ ACR-modified criteria] and 1.26 [JADAS-27]). The association between ESR and disease activity was also weak, with an OR of 1.11 [ACR-modified] and 1.13 [JADAS-27]. These findings did not reach statistical significance, although the 95% CI indicated good precision.

**Table 4:** Association between the four biomarkers and disease activity according to ACR-modified criteria and the JADAS-27 criteria.

	ACR-modified criteria (active disease)		JADAS-27 (active disease)	
	POR (95% CI)	*p*	POR (95% CI)	*p*
sCal EIA	1.52 (0.89–2.62)	0.12	1.79 (0.97–3.03)	0.06
SCal CLIA	1.19 (0.74–1.92)	0.46	1.26 (0.77–2.04)	0.35
ESR	1.11 (0.98–1.26)	0.08	1.13 (0.99–1.29)	0.06
CRP	1.79 (1.04–3.08)	0.03*	2.02 (1.12–3.65)	0.01*

POR, prevalence odds ratio; sCal, serum calprotectin; CLIA, chemiluminescence immunoassay; EIA, solid-phase enzyme immunoassay; CRP, C-reactive protein. ESR, erythrocyte sedimentation rate; CI, confidence interval.

*Statistical significance (*p* < 0.05).

Serum calprotectin levels measured by CLIA and EIA were strongly correlated (Kendall's tau-b, 0.71; *p* < 0.001). The scatter plot depicting this correlation is shown in Supplementary Figure S1.

#### 3.3.2 Multinomial logistic regression for JADAS-27

Both sCal EIA (ß=0.69, *p* = 0.04) and CRP (ß=0.74, *p* = 0.01) were significantly associated with high disease activity (JADAS-27 criteria). However, neither of these two biomarkers showed a significant association with mild disease (i.e., low activity) (Table 5).

**Table 5:** Correlation between the four biomarkers and disease activity according to JADAS-27 (mild, moderate, or high).

JADAS-27	CRP		ESR		sCal EIA		sCal CLIA	
	β (95% CI)	*p*	β (95% CI)	*p*	β (95% CI)	*p*	β (95% CI)	*p*
Mild	0.57 (-0.12–1.28)	0.10	0.07 (−0.12–0.26)	0.47	0.13 (−0.9–1.24)	0.81	0.13 (−0.97–0.92)	0.95
Moderate^a^	N/A	N/A	N/A	N/A	N/A	N/A	N/A	N/A
High	0.74 (0.13–1.34)	0.01^a^	0.13 (−0.01–0.27)	0.05	0.69 (0.01–1.36)	0.04^a^	0.28 (−0.22–0.79)	0.27

β, regression coefficient; CRP, C-reactive protein. ESR, erythrocyte sedimentation rate; sCal, serum calprotectin; CLIA, chemiluminescence immunoassay; EIA, solid-phase enzyme immunoassay; CI, confidence interval.

^a^ None of the patients presented moderate disease activity.

Neither sCal CLIA (ß=0.28, *p* = 0.27) nor ESR (ß=0.13, *p* = 0.05) were associated with high disease activity or mild disease activity as measured by JADAS-27 (ß=0.02, *p* = 0.95 and ß=0.07, *p* = 0.47, respectively).

## 4 Discussion

In the present proof-of-concept study, we aimed to assess the diagnostic accuracy of sCal measured by two different commercial assays, QUANTA Flash CLIA (Inova Diagnostics) and EIA (Bühlmann Laboratories). The ROC analysis based on JADAS-27 showed that the optimal cut-off value for disease activity was 2.3 µg/ml for both techniques. Remarkably, this threshold was consistent with the one established in our clinical practice. However, on the ROC analysis based on ACR-modified criteria, the optimal cut-off was 2.0 µg/ml for sCal CLIA—the same value recommended by the commercial kit—and 2.9 µg/ml for sCal EIA. By contrast, the optimal cut-off values for CRP and ESR (3.1 mg/L and 10 mm/h, respectively), according to both JADAS-27 and ACR-modified criteria were lower than the values commonly applied in routine clinical practice (5 mg/L and 20 mm/h, respectively). This finding demonstrates the limited sensitivity of these measures in identifying active disease when the standard thresholds are used.

When we used the ROC-derived cut-off of 10 mm/h for ESR (Tables 2, 3), the diagnostic accuracy was poor (both sensitivity and specificity <75%). However, when we applied the standard cut-off value (20 mm/h), the specificity was 100%, although sensitivity decreased (< 50%). For maximum efficiency, the optimal ESR cut-off was 20 mm/h, which is consistent with the values commonly used in routine clinical practice.

Serum calprotectin was more sensitive than both CRP and ESR (Tables 2, 3), although the specificity was lower. We compared the four biomarkers using both the JADAS-27 and ACR-modified criteria to assess disease activity. Based on JADAS-27, sCal EIA was more sensitive than sCal CLIA, CRP, and ESR. Based on the ACR-modified criteria, both sCal CLIA and EIA had a higher sensitivity than CRP and ESR. The higher sensitivity of sCal CLIA (ACR-modified criteria) suggests that this technique may be useful to evaluate extra-articular manifestations of JIA such as uveitis. Overall, in patients with active disease, sCal was more sensitive than CRP and ESR. For example, CRP and ESR were within the normal range (i.e., less than 5 mg/L or 20 mm/h, respectively) in more than 50% of patients with active disease. By contrast, sCal levels measured by EIA were only normal (i.e., below the cut-off for active disease) in two of 12 active patients (ACR-modified criteria) and 1 of 11 patients (JADAS-27). For sCal CLIA, the sCal values were considered normal in 5 of 12 patients (ACR-modified criteria) with active disease and 4 of 11 patients (JADAS-27).

At the time of data collection and biomarker assessment, one patient with active disease was experiencing an ongoing flare (simultaneous arthritis and uveitis). Although this patient presented elevated sCal levels (≥2.3 µg/ml)—indicative of active disease—there was no corresponding increase in either CRP or ESR, which suggests that sCal is better than CRP and ESR in identifying disease activity involving arthritis and/or uveitis, a finding that is consistent with prior research (6, 25). Alberdi et al. (12) recently examined the role of CRP as a predictor of long-term outcomes in JIA. In that study, CRP values >10 mg/L—which is double the standard cut-off value (5 mg/L)—were predictive of poor prognosis.

By contrast, in our study, both CRP and ESR had greater specificity than sCal at their standard thresholds, but a lower sensitivity. This lower sensitivity, together with the lower NPV, underscore the potential risk of these biomarkers to fail to identify disease activity in a substantial proportion of patients, a finding that is consistent with previous reports (6). In this regard, Sarkar et al. (26) found that CRP and ESR levels in patients with active JIA often remained below the cut-off levels for active disease, thus demonstrating that the presence of normal levels of these inflammatory markers does not necessarily indicate the absence of disease activity.

D'Angelo el al. (16). measured sCal using a different type of immunoenzymatic assay (Calprest NG from Eurospital Diagnostics). In that study, patients with active disease had higher sCal levels than those with inactive disease. That study also demonstrated a strong correlation between sCal and JADAS-27 (i.e., sCal levels were significantly elevated in active patients), a finding that is consistent with our results. Nonetheless, our findings suggest that sCal EIA may be less useful in differentiating between inactive and mild disease (low disease activity), as evidenced by the results of the multinomial regression analysis. By contrast to our results, D’Angelo et al. found that both CRP and ESR effectively differentiated between active and inactive disease.

In our study, both sCal EIA and CRP yielded similar results in terms of their capacity to identify the active disease (Tables 2, 3). By contrast, ESR was weaker (i.e., less able to distinguish disease activity), as evidenced by the lower sensitivity of this biomarker compared to the other three biomarkers. The lower sensitivity of ESR vs. CRP was somewhat surprising given that ESR—but not CRP—is a key component of the JADAS-27, the scale commonly used to assess disease activity status and to guide treatment decisions.

CLIA and EIA were closely correlated in terms of the quantification of sCal levels (Supplementary Figure S1), a finding that supports the reliability of these two techniques. Although the diagnostic accuracy (Table 3) of EIA was slightly better than CLIA (JADAS-27 criteria), the difference was not statistically significant.

One potential drawback of using sCal instead of CRP or ESR is the higher cost. However, sCal is probably more cost-effective than either CRP or ESR given the potential expenses related to suboptimal therapeutic decisions. Another potential disadvantage is the perception of CLIA and EIA as potentially more time-consuming than traditional biomarkers. Nevertheless, we found that turnaround time for both techniques was comparable to each other and similar to the immediate results obtained with traditional biomarkers. In this regard, it is worth noting that both of these assays are substantially faster than ELISA. An important advantage of CLIA over EIA is that CLIA is fully automated whereas EIA requires manual intervention.

### 4.1 Strengths and limitations

The main limitation of this proof-of-concept study is the small sample size (*n* = 25). Due to this limited sample, we were unable to perform multivariate or stratified analyses. Instead, we performed univariate regression analyses. However, we used non-parametric tests, which is a robust method to compare distributions without imposing specific distributional assumptions. Moreover, despite the small sample size, our findings were consistent with previous reports (6, 22).Another limitation is the single center study design, which could limit the generalizability of our findings. However, this limitation is mitigated in part by the type of hospital—a tertiary care hospital being a referral center for rheumatic diseases in Catalonia—and the large catchment area (> 450,000 inhabitants).

The main strength of this study is that it is the first to compare the diagnostic accuracy of two different methods (CLIA and EIA) to determine sCal levels and disease activity in a real-world population with JIA. Another strength is that we consistently followed a standardized protocol to measure sCal levels, thus minimizing potential variability, and that we have identified specific cut-off values to differentiate between active and inactive disease for both CLIA and EIA, thus providing clinicians with clear guidelines to accurately assess disease activity in real-world clinical settings.

The findings of this proof-of-concept study confirm the potential role of sCal as an inflammatory biomarker to monitor disease activity in patients with JIA. The prognostic value of sCal is notable, especially when remission status is unclear and there is a need to differentiate between inactive and active disease status. This is highly relevant given that both CRP and ESR often fall within the normal range despite the presence (or likely future development) of clinical signs of disease activity, such as uveitis or clinical arthritis (12, 27). In this regard, sCal could potentially serve as an indicator of the presence of mild persistent disease activity, even in the absence of clinical and/or biological indications of sustained inflammation. Moreover, sCal could also be used for the early detection of disease activity, which could have important clinical implications as it would allow clinicians to implement an effective treat-to-target strategy within a small window of opportunity, thus potentially preventing disease progression and/or complications (28). Similarly, determination of sCal levels could help to identify patients in treatment-supported remission who are likely to achieve sustained remission, thereby providing data that could support treatment discontinuation or tapering with minimal risk of relapse (29).

This study also shows that commercially available CLIA and EIA kits yield comparable sCal values, which supports the potential of both assays to evaluate disease activity in JIA patients. The analogous performance of these clinically-validated, commercially-available assays might enable clinicians to use this tool in routine clinical practice, in contrast to traditional biomarkers.

## 5 Conclusion

This study shows that determination of serum calprotectin levels, whether by CLIA or EIA, can accurately differentiate between active and inactive disease status, thus supporting the use of these assays in routine clinical practice. However, more studies are needed to better assess the diagnostic accuracy of sCal levels according to the specific JIA subtype. In particular, prospective studies are needed to evaluate the role of sCal to predict disease flares and treatment response. Ideally, multicenter studies should be performed to obtain more robust data and to confirm our findings across different patient cohorts and healthcare settings.

## Data Availability

The datasets presented in this study can be found in online repositories. The names of the repository/repositories and accession number(s) can be found in the article/**Supplementary Material**.
