# Supplementary material for: Diagnostic accuracy of serum calprotectin measured by CLIA and EIA in juvenile idiopathic arthritis: a proof-of-concept study
Source: Front Pediatr. 2024 Jun 19;12:1422916. doi: 10.3389/fped.2024.1422916 (PMC11219821; doi:10.3389/fped.2024.1422916)
Supplement: Supplementary file 1 [file Datasheet1.pdf]

Supplementary Material

1 Results of Bühlmann® EIA and QUANTA FLASH® CLIA

1.1 Diagnostic accuracy of sCal EIA, sCal CLIA, CRP and ESR for identifying disease activity

In our study population (n=25), we identified individuals who exhibited discordance between measurements of distinct inflammatory biomarker levels. Consequently, these measurements did not contribute significantly to confirming the patient's disease activity status (active or in remission) as determined by ACR-modified criteria and JADAS-27.

A total of seven patients had active disease (per ACR-modified criteria) and six had active disease (per JADAS-27), even CRP values were within the normal range (<5 mg/L) (Table 1A). Among the patients with active disease (ACR-modified's and/or JADAS-27 criteria), eight and seven individuals, respectively, had ESR levels below the 20 mm/h threshold (Table 1B). By contrast, only two patients with active disease (ACR-modified criteria) and one patient (JADAS-27) had sCal EIA levels below the 2.3µg/mL threshold for active disease (Table 1D).

Supplementary Table 1. Accuracy of sCal EIA, sCal CLIA, CRP and ESR for identifying disease activity

A

	ACR-modified			JADAS-27		
**CRP**	**Remission**	**Active**	**Total**	**Inactive**	**Active**	**Total**
Low (<5mg/L)	13	7	20	14	6	20
High (≥5mg/L)	0	5	5	0	5	5
Total	13	12	25	14	11	25

B

	ACR-modified			JADAS-27		
**ESR**	**Remission**	**Active**	**Total**	**Inactive**	**Active**	**Total**
Low (<20mm/h)	13	8	21	14	7	21
High (≥20mm/h)	0	4	4	0	4	4
Total	13	12	25	14	11	25

C

	ACR-modified			JADAS-27		
**sCal CLIA**	**Remission**	**Active**	**Total**	**Inactive**	**Active**	**Total**
Low (<2.3µg/mL)	7	5	12	8	4	12
High (≥2.3µg/mL)	6	7	13	6	7	13
Total	13	12	25	14	11	25

D

	ACR-modified			JADAS-27		
**sCal EIA**	**Remission**	**Active**	**Total**	**Inactive**	**Active**	**Total**
Low (<2.3µg/mL)	5	2	7	6	1	7
High (≥2.3µg/mL)	8	10	18	8	10	18

Total	13	12	25	14	11	25

Abbreviations: s*Cal, serum calprotectin; CRP, C-reactive protein; ESR, erythrocyte sedimentation rate*

1.2 Correlation between sCal levels measured by Bühlmann® EIA and QUANTA FLASH® CLIA techniques

After confirming the overall accuracy of sCal measurements, we evaluated the correlation of sCal measurements obtained by EIA and CLIA. As the figure below shows, the correlation was strong (Kendall's tau-b 0.71, p<0.001).

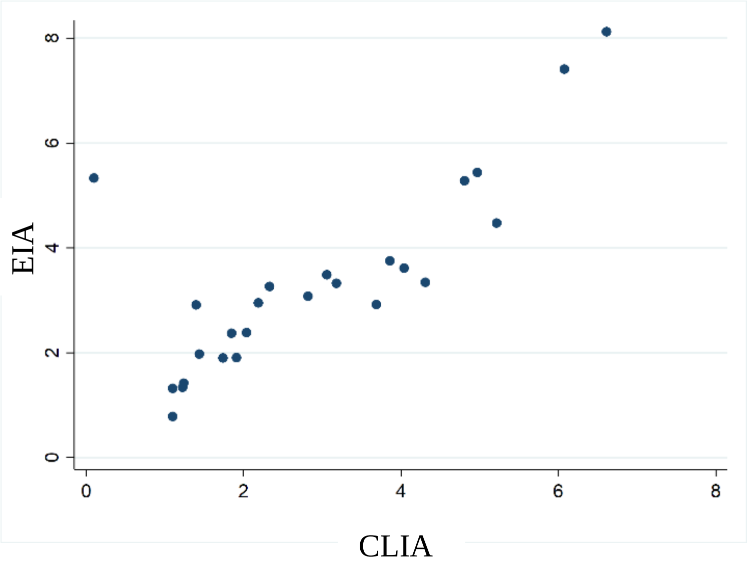

Supplementary Figure 2. Scatter plot showing the correlation between sCal EIA and sCal CLIA.
